# Vascular Endothelial Growth Factor (VEGF) and Its Role in the Cardiovascular System

**DOI:** 10.3390/biomedicines12051055

**Published:** 2024-05-10

**Authors:** Kamila Florek, Dominik Mendyka, Krzysztof Gomułka

**Affiliations:** 1 Student Scientific Group of Internal Medicine and Allergology, Wroclaw Medical University, 50-368 Wroclaw, Poland; 2 Department of Internal Medicine, Pneumology and Allergology, Wroclaw Medical University, 50-368 Wroclaw, Poland; krzysztof.gomulka@umed.wroc.pl

**Keywords:** ischemic heart disease, vascular endothelial growth factor, VEGF, anti-VEGF, tyrosine kinase receptors, cardiovascular disease

## Abstract

Cardiovascular diseases remain the leading cause of death worldwide, with ischemic heart disease (IHD) as the most common. Ischemia-induced angiogenesis is a process in which vascular endothelial growth factor (VEGF) plays a crucial role. To conduct research in the field of VEGF’s association in cardiovascular diseases, it is vital to understand its role in the physiological and pathological processes in the heart. VEGF-based therapies have demonstrated a promising role in preclinical studies. However, their potential in human therapies is currently under discussion. Furthermore, VEGF is considered a potential biomarker for collateral circulation assessment and heart failure (HF) mortality. Additionally, as VEGF is involved in angiogenesis, there is a need to elucidate the impact of VEGF-targeted therapies in terms of cardiovascular side effects.

## 1. Introduction

Cardiovascular diseases remain the leading cause of death worldwide [1]. Even though novel treatment options including medications and invasive treatment in that field have been developed, the background of ischemic heart disease (IHD) is still the most common [1]. Myocardial infarction (MI) may be the first manifestation of IHD [2]. It is crucial to mention that IHD is commonly defined as a state in which blood supply to the cardiac muscle is insufficient, which is mostly caused by atherosclerotic plaques formed in the coronary artery walls, as well as by blood clots or constriction [3]. However, according to the Fourth Universal Definition of Myocardial Infarction, MI is defined as prolonged ischemia resulting in myocardial cell death [4]. Moreover, the progression of IHD may lead to MI and, eventually, to subsequent post-MI heart failure (HF) development due to scar formation, cardiac muscle remodeling, and cardiac muscle hypertrophy [5]. Hence, concerning the role of vascular endothelial growth factor (VEGF), a focus on cardiac muscle circulation has to be made. Blood supply to the heart is provided by epicardial arteries (>500 µm), arterioles (100–500 µm), intramural arterioles (<100 µm), and capillaries (8–10 µm) [6,7]. Furthermore, microcirculation is defined as the terminal vascular network consisting of microvessels with diameters of <20 µm [8]. Microcirculation, due to its small wall thickness, forms an area where the oxygen and nutrients supply and consumption are balanced [9]. In health and disease, cardiovascular circulation adaptations occur—one of them is new blood vessel formation including collateralization with the involvement of certain processes. Namely, vasculogenesis occurs at the stage of embryogenesis when angioblasts migrate and form the endothelial cord and, prospectively, endocardial plexus; angiogenesis is stimulated by the ischemia; and arteriogenesis is associated mainly with arterial collaterals formation from pre-existing arteries, which happens mainly due to the occlusion or significant stenosis of the major arteries [10,11,12]. Angiogenesis stimulation can be physiological or therapeutic and it can accelerate collateral circulation formation and increase neovascularization [13]. Through other angiogenesis factors, VEGF is one of the most important and well-studied factors. This study aims to evaluate the role of VEGF in cardiovascular physiology and diseases. 

## 2. VEGF Role in Angiogenesis and Arteriogenesis

VEGF is one of the several particles involved in the process of endothelial cells and smooth muscle proliferation as well as migration. In angiogenesis, the role of VEGF is associated only with endothelial cell migration, whereas its involvement in arteriogenesis, including endothelial and smooth muscle cells, remains unclear [14,15]. The angiogenesis process is induced by hypoxia and arteriogenesis by increased shear stress [12,16]. However, previous findings suggest that only arterial capillaries may prevent myocardial ischemia with the co-presence of significant stenosis in major arteries [12,17]. When the epicardial artery is obstructed, the structural enlargement of arteriolar vessels and the formation of arterio-arterial anastomoses in the process of collateralization may be present as a compensatory mechanism [18]. Collateralization may provide ‘natural bypasses’ to the heart and prevent ischemic cardiac events [11]. Even though VEGF is mainly studied according to angiogenesis, several studies have proven the role of VEGF in cardiac muscle functioning and there are pathways describing VEGF’s involvement in the arteriogenesis process as well [19,20]. Additionally, VEGF plays an important role in physiological processes—it is crucial in fetal circulation development, and vessel development in the adult stage, as well as wound healing [21,22]. However, it is also involved in pathological pathways including tumor angiogenesis, atherosclerotic plaque neovascularization, and diabetic retinopathy [22,23].

The VEGF family consists of several factors expressed in humans—VEGF-A, VEGF-B, VEGF-C, VEGF-D, and placenta growth factor (PGF). Each one of these types has a distinct role and contributes to pathways by binding to specific tyrosine kinase receptors (TKR) named vascular endothelial growth factor receptors (VEGFRs)—VEGFR1, and VEGFR2—expressed mainly on the vascular endothelial cells (VECs), or VEGFR3 expressed on the lymphatic endothelial cells [24]. The main function of a particular VEGF is expressed by binding to several receptors. VEGFR1 activation leads to angiogenesis promotion and antiapoptotic and antioxidative effects. In addition, VEGFR2 shares with VEGFR1 proangiogenic and antioxidative functions, whereas VEGFR2 activation is associated also with an increased vascular permeability and proinflammatory mechanism of action. However, VEGFR3 stimulation increases lymphangiogenesis, fibrosis, and inflammation [25]. VEGF-A has an affinity to VEGFR1 and VEGFR2 and there are four variants of VEGF-A—121, 165, 189, and 206 based on the number of amino acids forming certain isoforms due to the alternative splicing of the VEGF-A gene consisting of 8 exons and 7 introns [26]. VEGF-B also activates VEGFR1 and VEGFR2 and two alternatively spliced isoforms have been identified—VEG-B167 and the predominantly expressed VEGF-B186 [27]. VEGF-C can bind to VEGFR3 and, with lower affinity, to VEGFR2; however, its mechanism of action is suspected to be level-dependent [25,28,29]. VEGF-D, similarly to VEGF-C, activates VEGFR2 and VEGFR3, but has a higher angiogenic potential and is not essential in lymphangiogenesis [26,30].

Angiogenesis consists of four stages: destabilization, sprouting, branching, and stabilization [12]. The molecular mechanism underlying endothelial cell proliferation is associated with cell metabolism including glycolysis and fatty acids oxygenation which produce substrates to stalk cell proliferation [31]. VEGF-A upregulation is induced by hypoxia and the subsequent increase in hypoxia-induced factor 1-alpha (HI-1α) binding to hypoxia-induced factor 1-beta (HIF-1β) leading to glycolysis [32]. The subsequent vasodilation, pericyte, and endothelial cell connection detachment are involved in the destabilization stage [32]. To promote the growth of stalk cells, the growth-inhibiting transcription factor forkhead box protein O1 (FOXO1) is inhibited by the phosphoinositide 3-kinase/protein kinase B (PI3K/AKT) in the pathway mediated by VEGF-A-VEGFR2 [31,32]. Then, by endothelial cell proliferation and tube-like structure formation, branching occurs [32]. Progenitor endothelial cells can be attracted via VEGF-B by cytokines’ granulocyte colony-stimulating factor, (G-CSF) and granulocyte-macrophage colony-stimulating factor (GM-CSF) [31]. The last stage of stabilization occurs with the basement membrane deposition over connected tubes and pericyte elongation over endothelial cells [31,32].

VEGF release in a neutrophil-dependent way occurs due to their recruitment in angiogenesis mediated by chemokine (C-X-C motif) ligand 1 (CXCL-1) and macrophage inflammatory protein-2 (MIP-2) [19]. Importantly, CXCL-1 administration also promotes arteriogenesis. It was also suggested that neuropilin 1 (NRP1) is significantly involved in arteriogenesis among adults as the loss of its cytoplasmic domain impairs arteriogenesis, whereas that domain promotes VEGFR2 endocytic trafficking [20].

Although VEGF involvement in angiogenesis is well-studied, its influence on arteriogenesis remains not wellunderstood. However, several studies are showing its role in heart function, including its contractility, repair of the cardiac muscle after MI, and prospective therapeutic target.

## 3. VEGF Involvement in Collateral Circulation Formation

In the newest meta-analysis, the collateral circulation impact on patients’ prognosis in MI, stable coronary artery disease (CAD), and restenosis were studied [33,34,35]. Patients who underwent acute MI and angiography had visible collateral circulation and had a more favorable prognosis according to long-term survival, as well as in terms of in-hospital mortality, and cardiogenic shock development [33,34,35]. Moreover, collateral circulation was revealed to be beneficial in patients with stable CAD as those who had highly developed collaterals had 36% lower all-cause mortality rates than those with lower collateralization [36]. However, the risk of in-stent restenosis after PCI was greater among patients with good collateral circulation [37]. In addition, another study revealed that some patients who underwent PCI and had chronic total occlusion of modified arteries had a poorer prognosis, although, in those cases, collateral circulation is said to increase the likelihood of a successful procedure [38]. The aforementioned data indicate that collateral circulation development should be favorable for certain groups of patients, namely, those with MI, CAD, and chronic total occlusions (CTOs). It is of note that VEGF’s roles have been studied in some of these circumstances. Despite the occurrence of proper reperfusion on the epicardial level, the unsatisfactory perfusion of cardiac muscle is observed in nearly 50% of patients. This phenomenon is caused by microcirculation dysfunction or obstruction and is associated with a negative prognosis [39,40,41]. Additionally, the role of VEGF is a topic of interest in studies investigating the potential use of this protein as a coadjuvant therapy among patients with MI [39]. Importantly, VEGF is increased during acute coronary syndromes. There is a study that showed that a higher VEGF-B167 concentration during acute MI was associated with higher monocyte CD14++CD16+ infiltration and the subsequent lower left ventricular remodeling [42].

Furthermore, interesting findings on collateral circulation formation were noted in animal studies. It has been shown that, in rats with induced diabetic mellitus type 2 (DM2), the combination of sodium butyrate administration and voluntary exercise after 8 weeks resulted in improved angiogenesis [43]. It is crucial as DM2 may be associated with the deprived development of collateral circulation [43]. In another study, vagus nerve stimulation (VNS) was revealed to be associated with increased repair by angiogenesis/arteriogenesis in hearts after myocardial infarction as its neurotransmitter acetylcholine increased VEGF-A and VEGF-B production via the m/nACh-R/PI3K/Akt/Sp1 pathway. However, VEGF-A showed a higher influence on endothelial cell tube formation as compared to VEGF-B, whereas the knockdown of both growth factors released an improving effect on left ventricle performance [44].

Moreover, VEGF involvement in coronary collateral circulation (CCC) was also studied by assessing its role as a biomarker of that process. It was revealed that, based on the Rentop grading system, VEGF-A was not associated with statistically significant differences between human groups with poor and well-developed CCC [44]. However, miRNA-146a was better at distinguishing those patients. Moreover, the newest meta-analysis concerning single nucleotide polymorphism (SNP) in human VEGF-A noted that rs699947, rs1570360, and rs3025039 polymorphisms were associated with CAD susceptibility and rs699947 and rs2010963 are biomarkers of poor CCC after myocardial ischemia [45]. Interestingly, VEGF-A rs2010963 polymorphism was associated with a higher risk of MI [46]. Additionally, a meta-analysis focused on a relationship between HF and SNPs in VEGF showed that SNP in rs748431 in FGD5 encoding the VEGF regulator was associated with a higher risk of rehospitalization and death [47].

## 4. VEGF as a Biomarker in Heart Diseases

VEGF is considered as a potential biomarker not only in terms of CCC formation but also according to the prognosis in different cardiovascular pathologies. It is crucial to mention that medications in CAD can be involved in VEGF concentration regulation. It has been shown that statins, in low doses, induce angiogenesis [48]. However, among diabetes mellitus patients, low doses of statins were revealed to decrease VEGF and basic fibroblast growth factor (bFGF) concentration in the serum, which suggests that there is no proangiogenic influence [48]. The aforementioned process is not the only studied interaction between statins and VEGF. It was shown that atorvastatin via the miRNA-221/VEGF-A axis stimulates the endothelial progenitor cell (EPC) proliferation, migratory capacity, and angiogenesis among patients with coronary slow flow (CSF) characterized by an impaired flow in the coronary artery without obstruction [49]. Moreover, VEGF-D was assessed as a biomarker predicting all-cause mortality among patients undergoing coronary angiography with suspected or known CAD [50].

**Figure 1:**
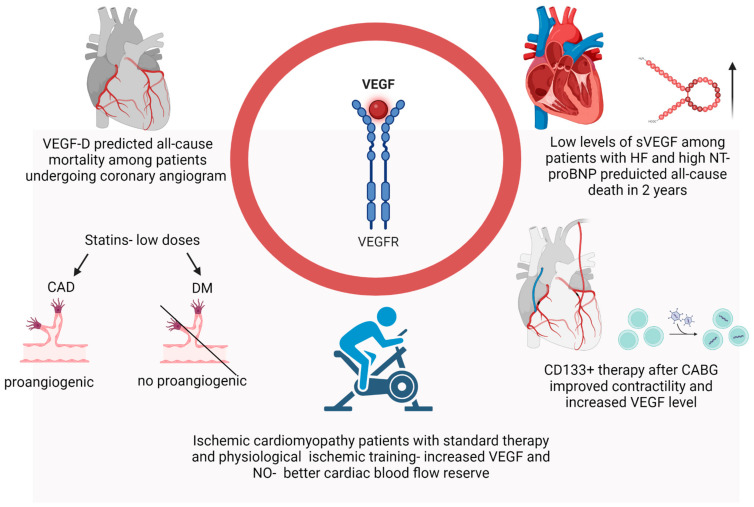
The role of VEGF as a biomarker. VEGF-D—a biomarker of all-cause mortality among patients undergoing coronary angiogram. VEGF is a biomarker of all-cause 2-year mortality among patients with HF and high NT-proBNP levels. The role of statins in regulating VEGF concentration, with varied effects observed in prognostication across different cardiovascular conditions. CD133+, as well as PIT and standard therapy, increased VEGF level.

Another study considering VEGF’s role as a biomarker was carried out in the field of HF [49]. It was a multicenter cohort study that revealed that low levels of soluble VEGF (sVEGF) were an independent prognostic factor of cardiovascular and all-cause death in two years, observed among patients with HF and high levels of NT-proBNP [49]. In addition, one randomized clinical study explored the role of CD133+ stem cell therapy in patients who underwent coronary artery bypass grafting (CABG) [50]. In the group after CABG with additional stem cell therapy, a significant improvement in cardiac muscle contractility with a concomitant increase in blood VEGF levels was observed [50]. A VEGF increase is suspected by authors to continue the improvement in myocardial perfusion when it is still not optimal [50]. Interestingly, another randomized clinical trial showed the positive impact of standard medical treatment with additional physiological ischemic training (PIT) on patients with ischemic cardiomyopathy. The conclusion of an improvement in the cardiac blood flow reserve was made based on the increased VEGF and NO in the peripheral blood in the group with PIT. In this group, lower cardiac remodeling and a renin–angiotensin–aldosterone system (RAS) were noticed as well [51] (Figure 1).

## 5. Clinical and Therapeutic Use of VEGF in Heart

Researchers have been investigating the use of VEGF as a therapy for ischemic heart disease since 1994. S. Takeshita described the beneficial effect of such a therapy on revascularization in the ischemic hindlimb of the rabbit, while S. Banai reported the successful generation of collateral circulation after the administration of recombinant human vascular endothelial growth factor (rhVEGF) in dogs in the same year [52]. In 1998 (Douglas W. Losordo), a study in five human patients showed a reduction in angina pain and nitrate use 30 days after the administration of rhVEGF for myocardial ischemia [52]. The therapy can be administered through the injection of the protein itself, DNA in the form of a plasmid, or mRNA. Researchers have been searching for the most appropriate way to deliver the protein or its precursor to the target cell. Various modes of VEGF administration have been studied, including intravenous, intracoronary, intramuscular transcatheter, or mini-thoracotomy routes. It is important to note that the choice of delivery method can impact the effectiveness of the therapy. In a study conducted by K. Sato and colleagues, the intravenous administration of VEGF in a pig model was found to be ineffective in eliciting a therapeutic response. However, promising outcomes were observed with coronary administration [53]. The safety and efficacy of the intravenous and intracoronary administration of rhVEGF-165 were evaluated in the VIVA study. The study revealed that the administration of rhVEGF-165 was well-tolerated over a 60- to 120-day follow-up period [54].

**Figure 2:**
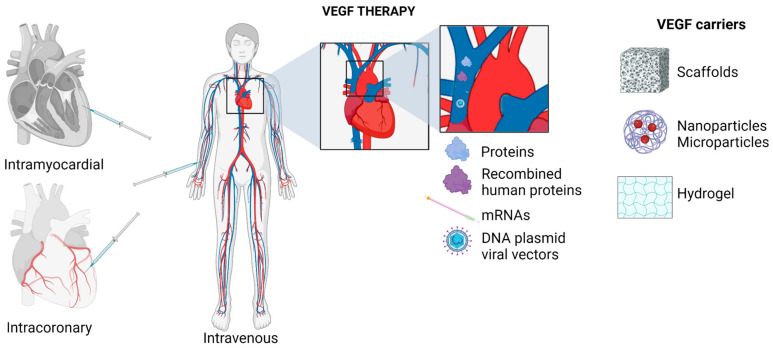
Routes of VEGF administration incorporate intramyocardial, intracoronary, and intravenous administration. The types of administered substances are proteins, recombined human proteins, mRNA, and DNA plasmid in viral vectors. The types of VEGF carriers are scaffolds, nanoparticles, microparticles, and hydrogel.

Various protein delivery platforms, such as scaffolds, nanoparticles, and microparticles, were considered in the context of VEGF protein delivery. However, the challenge is the rapid degradation of the peptide at the target site. The optimal approach would be to introduce the genetic material that encodes the desired protein into the cell. Currently, both viral and non-viral methods of vector transport are being studied. The most commonly used DNA plasmid among researchers is the recombinant type. However, its low transfection efficiency leads to a significant reduction in therapeutic properties. The EUROINJECT ONE trial administered an intracardiac injection of phVEGF-A-165 to patients with severe stable ischemic heart disease. The study showed a regional increase in wall mobility but did not demonstrate differences in stress-induced perfusion or an improvement in symptoms in the Canadian Cardiovascular Society (CCS) class [55] (Figure 2).

The NORTHERN study used plasmid DNA without a viral transmitter at a higher dose than in the previous study. However, single-photon emission tomography did not reveal any differences between the treated and placebo groups in terms of myocardial perfusion, treadmill exercise, or angina symptoms presented by patients [56]. The use of empty viral vectors is a significant breakthrough in scientific research. Adeno- and retroviral vectors are commonly used to transfer genetic material to postmitotic cells; even though they have their advantages, they may also cause serious immunological reactions. Studies have demonstrated the effectiveness of empty vector transfection. The second phase of the Kuopio Angiogenesis Trial (KAT) in 2003 showed that patients treated with adenoviral CMV-VEGF-165 during percutaneous coronary angioplasty had a statistically significant increase in myocardial perfusion after 6 months compared to those treated with VEGF-165 on lysosomal plasmid and Ringer’s lactate. The study showed that treating chronic myocardial ischemia with adenovirus-borne VEGF-165 had a positive effect on preventing postangioplasty and restenosis [57]. An 8-year follow-up found no significant difference in mortality or major adverse cardiac events (MACEs) between treatment groups. However, differences in cardiac symptoms, exercise tolerance, and the ability to work were observed [58].

The REVASC randomized controlled study utilized adenovirus-borne VEGF-121 and found a significant improvement in symptoms and exercise capacity in patients with nonvascularizable ischemic heart disease. During the treadmill exercise test, the ST segment decreased by 1 mm within six months. The requirement of thoracotomy in this particular study has been identified as a limitation due to its hindrance in the creation of a blank. The presence of this impediment has prevented the researchers from carrying out the necessary procedures to establish an adequate control group. As a result, this study’s findings may be limited in their capacity to provide a complete understanding of the subject matter, and it may be necessary to devise alternative methods to address this limitation in future research [59]. The therapeutic gene, XC001, which expresses the three main vascular endothelial growth factor isoforms, has demonstrated an improved angiogenic response and enhanced safety by upregulating the relative expression of VEGF-189. The therapy has exhibited promising outcomes in animal studies, particularly in rat hearts, and has subsequently been granted permission for clinical trials in humans [60]. The EXACT Trial is a study that has optimized the use of the XC001 gene to preferentially express the heparin-binding VEGF-189 and VEGF-165. Follow-up after a year has revealed that higher doses have led to greater increases in the overall exercise time on the treadmill and reductions in ischemia and CCS angina compared to lower doses [61].

VEGF is commonly used in combination therapies. In a study on patients with severe chronic ischemic heart disease, Ripa and his research group proposed a combination of VEGF-165 with G-CSF. However, the expected effectiveness was not demonstrated, and they proposed increasing the dose [62]. The clinical study, VIF-CAD, employed a combined therapy to treat patients suffering from refractory heart ischemia. The plasmid used in the study combined VEGF with fibroblast growth factor (FGF) for the treatment. The study results indicated that this therapy was safe for the patients. Although there were no significant differences in perfusion changes between the study group and the placebo, it demonstrated notable improvement in the patient’s condition. The study showed a slight increase in treadmill running time and a significant improvement in maximum workload, total test distance, and CCS class during the 5- and 12-month follow-up. Thus, the authors concluded that, despite the lack of increased perfusion, this therapy could reduce clinical symptoms and improve the patient’s condition [63]. Recent scientific reports suggest the beneficial use of injecting naked informative RNA (mRNA) encoding VEGF-A directly into the heart muscle. A study named EPICCURE, published in 2022, demonstrated that AZD8601, an mRNA therapy formulated in citrate saline buffer without lipid encapsulation for local administration in patients undergoing CABG, met the primary endpoint of safety and tolerability in patients with heart failure. Positive results were observed in three categories: LVEF, NT-proBNP, and functional patient outcomes [64].

In the context of scientific literature, VEGF-D is frequently mentioned alongside VEGF-A. A 2017 study, identified by plate number KAT301, sought to evaluate the safety, myocardial perfusion, angina pectoris, and quality of life of refractory angina patients who received targeted intramyocardial gene VEGF-DΔNΔC therapy with an adenoviral vector. The one-year follow-up conducted by the study demonstrated that the treatment was safe, with the only concern being the production of adenoviral antibodies that could potentially hinder the delivery of genes to the cell if a subsequent dose is necessary. The study showed significant improvements in CCS class and quality of life, and the objective of long-term improvement in the treated areas was also noted. Moreover, the study recommended using elevated plasma Lp(a) as a biomarker to identify patients who may benefit from AdVEGF-DΔNΔC gene therapy [65]. During the 8-year monitoring period, no complications associated with the administration of VEGF-D were reported. However, there were apprehensions regarding the systemic distribution of VEGF and the potential impact of VEGF-D therapy on the progression of latent cancer via diverse signaling pathways, specifically in the liver. Given that the liver is accountable for vector removal, it was crucial that we further investigate the potential impact of VEGF-D therapy on cancer progression [66].

## 6. VEGF Inhibition in Therapies and Their Influence on Cardiovascular Outcomes

**Figure 3:**
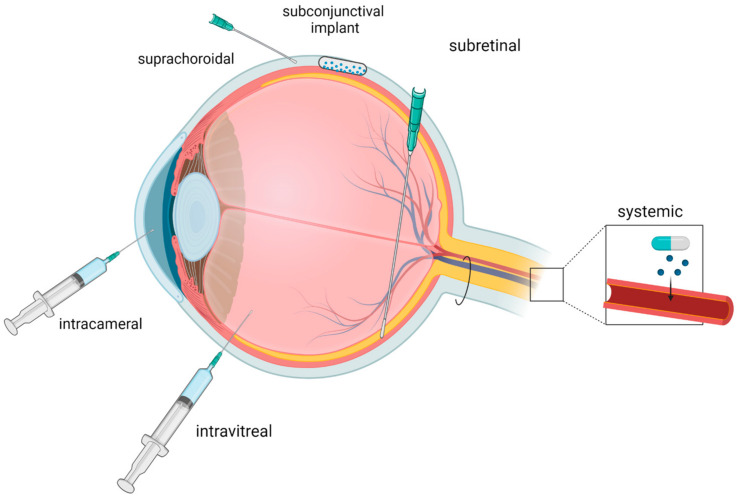
VEGF inhibitors delivery methods. The most widely used in ophthalmology—anti-VEGF intravitreal injections, intracameral, suprachoroidal administered with the microcapillaries, subretinal, and subconjunctival implants, which are at the phase III trial [68,69,70]. Oral administration of VEGF inhibitor medications is mainly used in cancer treatment and is related to the highest concentration in the systemic circulation.

Even though VEGF is studied as a therapeutic factor in cardiovascular diseases, nevertheless, its inhibition is considered beneficial in oncology and ophthalmology. Vascular endothelial growth factors tyrosine kinase inhibitors (VEGF-TKIs) are used in cancer therapy and anti-VEGF treatment is extensively studied according to the topic of retinal vein occlusion, diabetic retinopathy (DR), macular degeneration, and neovascular glaucoma. Referring to cancer treatment, VEGF-TKIs are mainly available for oral administration, whereas the delivery methods in terms of anti-VEGF treatment in ophthalmology are more diverse. These methods differ in their efficacy and safety; thus, it is vital to consider those parameters while choosing the optimal method of drug delivery. Moreover, distinct delivery methods are associated with various probabilities of a systemic effect. A systemic administration due to the blood–retinal barrier reveals a low—1–2%—drug concentration in the vitreous humor and a higher potential risk of systemic adverse effects [67]. Other methods of anti-VEGF treatment are intravitreal, intracameral, subretinal, or suprachoroidal injections [67,68]. Furthermore, throughout the scleral incision, it is possible to place the implant which provides a sustained release of the anti-VEGF medications [69,70]. Currently, anti-VEGF agents are administered mainly intravitreally which reduces systemic side effects but is related to various complications ranging from subconjunctival hemorrhage to rhegmatogenous retinal detachment [71] (Figure 3).

These contrary beneficial pathways suggest examining if there are any side effects of widely used anti-VEGF therapies on the cardiovascular system. It is crucial that we highlight the results of the most recent meta-analysis exploring whether there is an influence of VEGF-inhibiting therapies on the cardiovascular system. The findings suggest that VEGF-TKIs in different malignancies have an impact on the risk of certain cardiovascular consequences—MACEs, and heart failure, as well as thromboembolism [72]. The risk of several endpoints was different for specific VEGF-TKIs; however, this study concluded that higher-potency and lower-sensitivity therapeutics increased HF events. High potency alone was associated with a greater probability of MACEs, and the lower the selectivity, the higher the risk of thromboembolism [72]. Those findings identifying particular medications should be taken under consideration while treating patients with malignancies in the decision-making scheme. Another meta-analysis investigated the role of VEGF-TKIs in solid tumor treatment and their cardiotoxicity potential [73]. They identified that some medications did not show cardiotoxic outcomes—regorafenib and nintedanib—whereas lenvatinib and vandetanib had the most severe cardiotoxic impact [73]. Lenvatinib was also highlighted in the previously mentioned study as the second-most potent drug according to MACE risk, following tivozanib [72]. Moreover, lenvatinib was the strongest predictor of thromboembolism [72]. There was also a systematic review published considering cardiovascular adverse events in anti-VEGF oncological treatment alone vs. its use in combined therapy with immune checkpoint inhibitors (ICIs) [74]. The anti-VEGF with ICI treatment was associated with an increased risk of cardiovascular toxicity [74].

A cohort study among patients with DR and intravitreal anti-VEGF injection (IAVI) therapy has shown that, in comparison to laser or steroid treatment, there were no significant differences in the risk of cardiovascular events [75]. Another cohort study showed that IAVIs among patients with diabetes increased the risk of cardiovascular disease events; however, it was not statistically significant [76]. There are a lack of data from randomized clinical trials focusing on the influence of anti-VEGF treatment on cardiovascular adverse events. There is a meta-analysis showing that IAVIs in retinal vein occlusion did not increase the risk of cardiovascular events, hypertension, or heart rate disorders [76]. Moreover, according to the most recent meta-analysis, continuous positive airway pressure (CPAP) in patients with obstructive sleep apnea (OSA) failed to reduce cardiovascular events [77]. As compared to the group with the usual treatment, CPAP was related to an increase in proinflammatory lung distension-responsive angiopoietin-2 and a decrease in cardioprotective VEGF-A [78]. Those data according to CPAP form an interesting research field for VEGF-based therapies. VEGF-targeted therapies are excessively studied in oncology and ophthalmology, and their systemic influence is highlighted as well. Hence, it is crucial to identify the safest medications and delivery methods for patients with several comorbidities, as well as the use of these medications that have the lowest side-effect potential.

### 6.1. Recent Clinical Trials Considering VEGF Inhibitors in Terms of Cardiovascular Complications

There are not enough data from clinical trials focusing on the influence of anti-VEGF treatment on cardiovascular adverse events. The only medications considered in the clinical trials were brolicizumab, sunitinib, and sorafenib, as well as cediranib in combination with selumetinib [79,80,81]. Brolicizumab intravitreal injections in neovascular age-related macular degeneration (nAMD) did not significantly influence cardiac pathologies. However, the methodology of assessment of cardiovascular safety is only by electrocardiogram (ECG) which does not provide information about the majority of the aforementioned cardiovascular complications [79]. Data from the ASSURE trial were analyzed among adolescents and young adults (AYAs) in terms of cardiovascular function assessed by the left ventricular systolic diameter and hypertension. Those patients were treated with sunitinib or sorafenib. The results showed that sunitinib was associated with a significantly lower incidence of hypertension as compared to sorafenib. Additionally, hypertension after treatment with those medications was less common among the AYA than non-AYA group [80]. Moreover, results from a phase I trial on cediranib in combination with selumetinib use in solid tumors showed that continuous therapy was not well-tolerated in terms of cardiovascular toxicities. Those that were possibly associated with treatment were decreased LVEF, palpitations, sinus tachycardia, prolonged QTc, and hypertension [81] (Table 1).

### 6.2. Mechanisms Underlying Cardiovascular Effects

The mechanisms underlying the aforementioned potential cardiovascular effects are associated with the inhibition of VEGF function. Firstly, VEGF-induced nitric oxide (NO) production is inhibited, which suppresses NO-dependent vasodilation, antithromboticity, and angiogenesis. This clinically raises the risk of myocardial infarctions, strokes, venous thromboembolisms, and hypertension [82]. Furthermore, two of the most widely used anti-VEGF medications—ranibizumab and aflibercept—were confirmed in in vitro studies to increase the proinflammatory agent’s concentration, playing a crucial role in atherosclerotic plaque formation [83]. Thus, patients with CAD and ischemic HF should be under higher vigilance during the treatment or other medications with a lower proatherogenic potential should be considered. Moreover, proinflammatory conditions, as well as VEGF-A inhibition, which displays an anti-fibrotic role, may increase fibrosis, highering the risk of HF [84]. VEGF-inhibiting therapies in cancer treatment are associated with cardiotoxicity leading to cardiomyocyte loss or a lower repair capacity. The most convincing mechanism of VEGF-TKI- is associated with the activation of the cardiomyocyte mitochondria-induced apoptosis pathway [85]. As intraocular injections are associated with subsequent lower systemic concentrations than with oral administration in cancer treatment, those side effects may be less probable. However, cardiotoxicity is widely studied in terms of VEGF-TKI treatment; it should be elucidated in ophthalmologic patients as well, as there are no concise data about such effects. Moreover, the maximization of therapeutic benefits while mitigating the cardiovascular risks would be possible by a tailor-made approach in VEGF-therapy choosing and by a certain cardiological surveillance strategy development. The individual approach should consider patients’ comorbidities, and cardiovascular risk factors, with a correlation to drug potency and selectivity. In addition, the drug administration route matters and strategies associated with the lowest systemic concentrations should be favorable.

## 7. Discussion

VEGF’s role in angiogenesis is crucial; however, its role in arteriogenesis is still unclear. VEGF therapy has been extensively studied in scientific publications and clinical trials, revealing both advantages and disadvantages. Animal models, including rabbits, dogs, and preliminary human studies, have demonstrated the potential efficacy of VEGF therapy in promoting revascularization and alleviating angina symptoms in ischaemic conditions. However, a significant challenge in VEGF therapy is the rapid degradation of the VEGF peptide at the target site. To overcome this challenge, various delivery methods are under investigation, including DNA plasmids containing single genes, as well as multiple VEGF isoforms and mRNA, which show promise in terms of safety. It is important to note that the mode of delivery can significantly impact the efficacy of the therapy. Among the available delivery methods, empty viral vectors, particularly adenoviral vectors, have shown efficacy in delivering VEGF genes. In addition, combination therapy with other growth factors or cytokines has been investigated to enhance the effectiveness of VEGF therapy. However, certain combination therapies, such as VEGF-165 with G-CSF, have not demonstrated the anticipated efficacy, while others, such as the combination of VEGF with FGF, have exhibited significant improvements in patient condition and clinical symptoms. Furthermore, VEGF is considered a promising biomarker according to HF and to assess the mortality risk among patients undergoing coronary angiography. In addition, the influence of statins on VEGF level regulation is vital. Moreover, in the era of extensive research about VEGF-targeted medications, it is necessary to define its systemic impact, especially according to the side effect in the field of cardiovascular diseases.

## 8. Limitations

According to the limitations and challenges of previously mentioned therapies, it is crucial to identify the most efficient delivery methods of VEGF peptide and search whether there are efficient co-therapies considering other growth factors. Moreover, there is a need to elucidate in dedicated studies VEGF-inhibiting therapies’ role in the development of certain cardiovascular complications and create concise guidelines on how to choose an optimal medical treatment and monitor patients who are at high cardiovascular risk.

## 9. Conclusions

Despite the potential of VEGF therapy in treating ischemia, further research is necessary to redefine dosing strategies, improve delivery methods, and address potential safety concerns, particularly regarding long-term effects and combination therapies. Therefore, ongoing research in this area is a critical step in advancing the field and improving the treatment of ischaemic conditions. Furthermore, VEGF’s role as a biomarker in the processes and diseases associated with the heart is needed. Currently, it is crucial that we focus on defining the safest medications among VEGF-targeted therapies with the lowest probability of cardiovascular side effects.

## Figures and Tables

**Table 1:** Recent clinical trials considering VEGF inhibitors in terms of cardiovascular complications.

Clinical Trial	Medication	Chemical Structure	Results
BEL study [79]	Brolicizumab	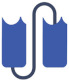	Based on the ECG parameters, brolicizumab intravitreal injections did not cause cardiovascular complications.
ASSURE trial [80]	sunitinib or sorafenib	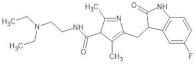 sunitinib 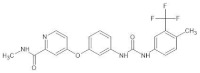 sorafenib	Sunitinib was associated with a significantly lower incidence of hypertension than sorafenib.
Phase I study by Hubbard et al. [81]	cediranib and selumetinib	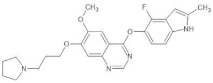 cediranib 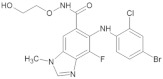 selumetinib	The continuous therapy with both drugs was not well-tolerated in terms of cardiovascular toxicity. Intermittent schedules may be needed.
